# The effects of physical activity on executive function in children with attention-deficit/hyperactivity disorder: A systematic review and meta-analysis protocol

**DOI:** 10.1097/MD.0000000000015097

**Published:** 2019-04-05

**Authors:** Mei-Qi Zhang, Zhan Liu, Hong-Tao Ma, Dong Zhang

**Affiliations:** a Springfield College, MA; b Beijing Sport University, Beijing, China.

**Keywords:** ADHD, children, cognition, executive function, physical activity

## Abstract

**Background:**

The effects of physical activity on executive function are well documented, but whether physical activity contributes to the executive function of attention deficit hyperactivity disorder (ADHD) children are still inconclusive.

**Methods:**

The study is guided by the Preferred Reporting Items for Systematic Review and Meta-analysis Protocols (PRISMA-P). We will search the following databases PubMed, EMBASES, the Cochrane Library, CNKI, and Wanfang-Data to identify the Randomized Controlled Trials evaluating the effects of physical activity on executive function among ADHD children. The language of literature restricted in Chinese and English, which published from inception to January 2019. Two reviewers will screen the studies independently, while risk of bias assessment, data extraction, and inconsistent results will be discussed by the third reviewer. Revman 5.3 and Stata 12 software will be used to complete data analysis and synthesis.

**Conclusion:**

This study will be based on findings of previous studies, thus the ethics approval is not required. The final results will be presented at an international conference and submitted to a peer-reviewed journal of relative field for consideration of publication.

**PROSPERO registration number:**

CRD42019118622.

## 1 Introduction

Attention deficit hyperactivity disorder (ADHD) is a psychiatric neurodevelopmental disorder^[1–3]^ that is sometimes found in children over 6 years old. Children with ADHS display symptoms such as difficulty paying attention, excessive activity, or difficulty controlling behavior which are not appropriate for their ages.^[4]^ Epidemiological studies have shown that ADHD affected about 7% to 11%^[5,6]^ of children worldwide, which has attracted more attention in recent research. ADHD is considered as a significant detriment to the quality of life with known symptoms^[7,8]^, and evidences have shown that these symptoms could lead to poor school performance and even persist in later life.^[9–11]^

Usually, the conditions of ADHD are related mainly to 2 crucial components: cognitive and behavioral problems.^[12]^ Executive function is the most important part of cognition which is associated with self-regulatory cognitive processes, such as monitoring and controlling both thought and goal-directed behaviors. The detrimental effects of poor executive function, especially working memory, set-shifting, inhibition, attention, and updating can lead not only to poor academic performance, but also to lifelong difficulties in family, social life, employment, and communication functioning.

Physical activity is an important component in manifesting cognitive function, and it has been recommended by the Centers of Disease Control and Prevention (CDC) that children should participate in at least 60 minutes per day of moderate to vigorous intensity physical activity (MVPA) and at least 3 times per week of high-intensity exercise.^[13]^ Previous researchers have found that physical activity is an effective method to improve executive function including attention, working memory, planning, and problem-solving.^[14–16]^ However, less amount of attention has been directed to the study of physical activity intervention in children with ADHD than that to normal children. It still remains unclear whether physical activity is an effective method in enhancing the executive function of children with ADHD and how physical activity affects their executive function. Therefore, the purpose of this study is to explore the effects of physical activity on executive function in children with ADHD. To this end, the proposed study will answer the following questions:

- Does physical activity improves executive function in children with ADHD?
- Does physical activity intervention have different effects on specific components of executive function?
- Can a subgroup analysis identify the effects of physical activity on executive function in children with ADHD?

## 2 Methods

This review protocol has been registered in the PROSPERO international prospective register of systematic reviews, the registration number: CRD42019118622. The Preferred Reporting Items for Systematic Review and Meta-analysis Protocols (PRISMA-P)^[17]^ will serve as guidelines for reporting present protocol and subsequent formal meta-analysis.

### 2.1 Search strategy

To avoid missing any available literature that is possible to meet our demands, we will comprehensively search electronic databases, including PubMed, EMBASES, the Cochrane Library, CNKI, and Wanfang-Data. All the English and Chinese literature will be sought. Search strategy will be built according to guidance from the Cochrane handbook. Search strategy for PubMed is shown in Table 1, and similar strategies will be built and applied for other databases.

**Table 1:**
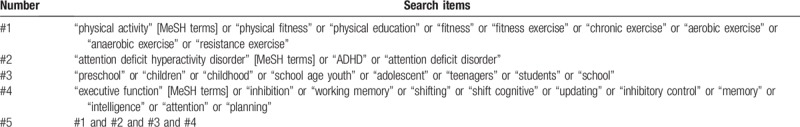
Search strategy in PubMed.

### 2.2 Inclusion and exclusion criteria

#### 2.2.1 Study design

We will only include randomized controlled trials (RCTs), whereas crossover trials, quasi-RCTs, and non-RCTs will not be eligible for inclusion.

#### 2.2.2 Participants

Referring to the definition^[4]^ of ADHD, we will include only studies in which children and adolescents with ADHD were recruited as participants. In particular, we will include studies in which participants were diagnosed with ADHD by a medical professional.

#### 2.2.3 Intervention

We will accept all types of physical activity intervention studies without any restrictions about their training method, duration, frequency, intensity. In these studies, the experimental group should receive by physical activity/exercise combining or not combining other treatment; while the control group should be treated by sedentary resting, or no exercise/treatment. Some other treatment interventions (e.g., medicine) are selectively acceptable.

#### 2.2.4 Outcome measures

In our study, all measurements of suitable studies will be considered as outcomes. The effects of physical activity for ADHD children's executive function are mainly evaluated by the changes between pre and post intervention. For the purpose of this review, we will involve all outcome measures used to assess executive function that are reported by researchers in the included studies (e.g., Flanker task, N-back task, Stroop test, Go/No go task, and Wechsler Intelligence Scale for Children 4th edition). The secondary outcomes mainly involve adverse events, adherence to intervention, and patient-reported outcomes.

### 2.3 Studies selection and data extraction

**Figure 1:**
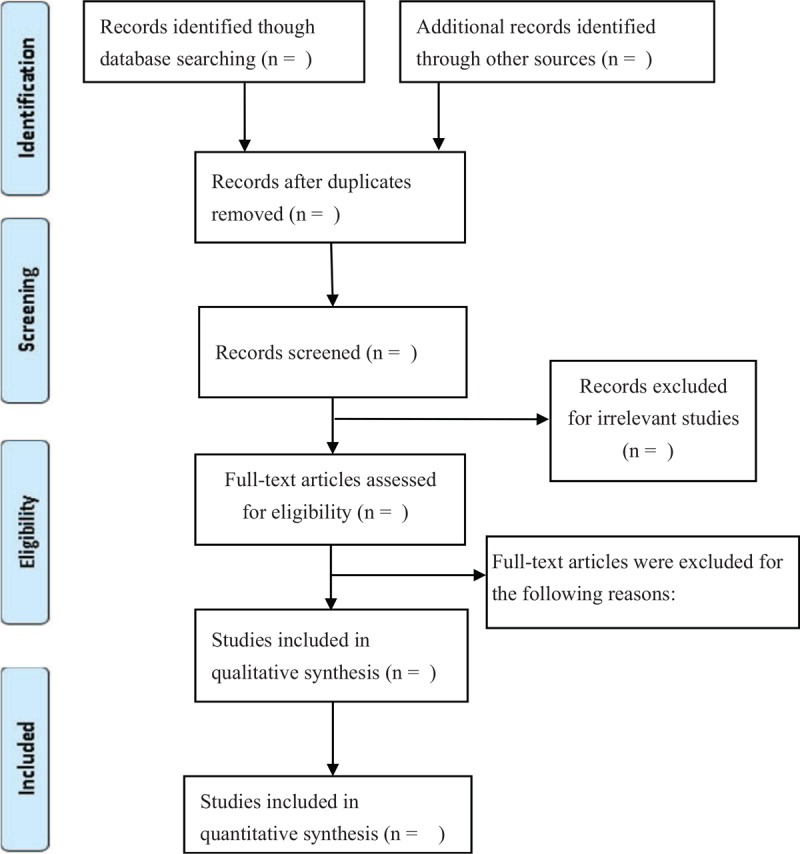
Flowchart of selecting progress. Included study will accord with the PRISMA statement. We will get rid of same studies form different databases by evaluating titles and abstracts, and then evaluate full-texts of the eligible studies. Finally, we will conduct qualitative or quantitative evaluation. (Moher D, Liberati A, Tetzlaff J, Altman DG, The PRISMA Group (2009). Preferred Reporting Items for Systematic Reviews and Meta-Analyses: The PRISMA Statement. PLoS Med 6(7): e1000097. doi:10.1371/journal. pmed 1000097.)

For the initial screening stage, the title and abstract of literature will be selected independently by 2 review authors. Recording and managing related literature will use Endnote software. Second, full texts of selected studies, the 2 authors will check suitable studies with the inclusion criteria. Finally, the 2 authors will put their screened studies together and include studies will be determined. Disagreement will be solved by discussion, and then make a consultation with other authors. The selection process of suitable literature will be shown in the Preferred Reporting Items for Systematic Review and Meta-analysis (PRISMA) flowchart (Fig. 1).

### 2.4 Risk of bias assessment and Grading of Recommendations Assessment, Development, and Evaluation (GRADE) assessment

The Cochrane Collaboration's tools will be used to check the random sequence generation, allocation concealment, blinding, incomplete outcome data, selective reporting, and other bias, each of them will be determined as a high-risk, low-risk, or unclear grade by 2 independent reviewers. The GRADE system will be used to appraise the quality of the evidence by 2 independent reviewers. The grating of evidence quality and risk of bias by 2 reviewers will be compared, and the third reviewer will be contacted if the consensus is not attained.

### 2.5 Data analysis and synthesis

If it is possible to conduct a meta-analysis, the Review management software Revman 5.3 and Stata 12.0 will be used. The continuous outcomes will be expressed as mean difference (MD) with 95% confidence interval. When the MD of the outcomes is large or the unit is different, the standardized mean difference (SMD) will be used. The heterogeneity test between studies will be assessed with the Q statistic and the I^2^ statistic. Usually, if *P* >.01, I^2^ <50%, it will be considered that heterogeneity is low enough and a meta-analysis can be conducted with a fixed-effect model. If *P* <.10, I^2^ >50%, it will be considered as high level of heterogeneity, and a random effect model will be used. Sensitivity analysis will be used to further reducing heterogeneity by removing studies with high risk of bias or individually omitting each study to explore the sources of heterogeneity. Sources of heterogeneity will be investigated by meta-regression using age, gender, exercise intensity, and duration of exercise as covariates. Last, funnel plots and Egger test will be used to assess the publication bias.

### 2.6 Subgroup analyses

Subgroup analyses will be performed on physical activity/exercise type (e.g., aerobic exercise, resistance exercise, motor skill training, or mixed training), intensity, frequency and/or duration, and so on. The purpose of subgroup analyses is to determine the best physical activity form of ADHD children.

## 3 Results and discussion

To the best of our knowledge, this will be the first systematic review and meta-analysis protocol to focus the effects of physical activity interventions on executive function in children with ADHD. Completing this protocol will determine if physical activity is a superior modality for children with ADHD in improving their executive function. Lastly, we believe that this study may lead to several recommendations, for parents, teachers, pediatric doctors, and researchers, such as what kind of exercise method is the best and which exercise intensity is more suitable for children with ADHD.

## Author contributions

Meiqi Zhang contributed to study conception and design, drafting the submitted article, and critically revising the draft for important intellectual content. Dr Zhan Liu revised the draft critically for important intellectual content and gave final approval of the version for publication. Dr Hongtao Ma and Dong Zhang contributed to acquisition, analysis, and interpretation of the data.

**Data curation:** Dong Zhang.

**Project administration:** Hong-Tao Ma.

**Writing – original draft:** Meiqi Zhang.

**Writing – review & editing:** Zhan Liu.
